# The association of widowhood and living alone with depression among older adults in India

**DOI:** 10.1038/s41598-021-01238-x

**Published:** 2021-11-04

**Authors:** Shobhit Srivastava, Paramita Debnath, Neha Shri, T. Muhammad

**Affiliations:** International Institute for Population Sciences, Mumbai, Maharashtra 400088 India

**Keywords:** Geriatrics, Public health

## Abstract

Widowhood is a catastrophic event at any stage of life for the surviving partner particularly in old age, with serious repercussions on their physical, economic, and emotional well-being. This study investigates the association of marital status and living arrangement with depression among older adults. Additionally, the study aims to evaluate the effects of factors such as socio-economic conditions and other health problems contributing to the risk of depression among older adults in India. This study utilizes data from the nationally representative Longitudinal Ageing Study in India (LASI-2017–18). The effective sample size was 30,639 older adults aged 60 years and above. Descriptive statistics and bivariate analysis have been performed to determine the prevalence of depression. Further, binary logistic regression analysis was conducted to study the association between marital status and living arrangement on depression among older adults in India. Overall, around nine percent of the older adults suffered from depression. 10.3% of the widowed (currently married: 7.8%) and 13.6% of the older adults who were living alone suffered from depression. Further, 8.4% of the respondents who were co-residing with someone were suffering from depression. Widowed older adults were 34% more likely to be depressed than currently married counterparts [AOR: 1.34, CI 1.2–1.49]. Similarly, respondents who lived alone were 16% more likely to be depressed compared to their counterparts [AOR: 1.16; CI 1.02, 1.40]. Older adults who were widowed and living alone were 56% more likely to suffer from depression [AOR: 1.56; CI 1.28, 1.91] in reference to older adults who were currently married and co-residing. The study shows vulnerability of widowed older adults who are living alone and among those who had lack of socio-economic resources and face poor health status. The study can be used to target outreach programs and service delivery for the older adults who are living alone or widowed and suffering from depression.

## Introduction

The pace of world population aging has been the fastest in recent years. According to Help Age India, the people aged 60 and above are classified as older population in India, whose proportion is projected to rise to 19.5 percent by 2050^1^. However, the significance of the rapid increase in life expectancy and its implication is yet to be appreciated as increased life expectancy does not mean the improved quality of life among older adults^2^. Most countries, including developing countries with a constant increase in longevity, pose serious challenges such as increased risks of multi-morbidity, disability, dementia and, weakening their functional capability, and increasing dependence for personal, household, and medical care^3,4^.

The average life span in the world is expected to double in the twentieth century^5^. With increased longevity in some countries, the concept of widowhood has broadened due to growing concerns of older adults whose spouse has died, and their likelihood of living alone for a longer period has also increased^6^. Approximately 350 million people are estimated to be widowed in 2020, out of which about 80 percent are females^7^. Some predominant reasons for increased risk for women to outlive men are related to the higher life expectancy of females due to their biological advantages over men. However, the differences in life expectancy between the two sexes are not purely biological and there are intervening social factors such as the age gap in marriage, higher risk-taking behavior, and increased substance use among men^8^.

Widowhood is an irreversible situation^9^. It is a catastrophic event at any stage of life for the surviving partner with serious repercussions on their physical, economic, and emotional well-being, particularly in the first year of the loss or for a longer term in some cases^10^. However, the emotional response to the spousal loss is hypothesized to be different depending on various socio-demographic characteristics such as age, gender, widowhood duration, living arrangement, functional ability to perform activities, health status and other factors such as community involvement and economic conditions of the survivor^11–13^. Widowhood often places individuals at a greater risk of deteriorating health and depressive symptoms with passing time post spousal loss for both sexes^7^. Across studies, prevalence rates of depressive symptoms are estimated to be high as 15–30 percent within the first year of widowhood^10^. However, it remains unclear whether widowhood has more psychological consequences on males or females. Differences between the two sexes in depression due to spousal loss is argued to differ according to gender roles; as compared to men, women are found to invest less in their financial security and more in familial relationships. After the bereavement of the spouse, their only source of income is lost, which increases their economic hardships at older ages leading to an adverse impact on their psychological well-being^7,14,15^. Few studies suggest that men and women have similar difficulty adapting to widowhood and reorganizing their lives after the spousal loss^6^. However, the loss of a spouse is not always associated with negative outcomes. For instance, some studies observed improvement of well-being and lower depressive symptoms among older widows after spousal bereavement, due to reorientation of life, and increased time for individual development because of the decreased burden of caregiving^16^. Considering the large variation in reaction to spousal loss, this as a generalized hypothesis as an outcome of widowhood is not appropriate. In many countries like India where widows account for a larger proportion of the population, widowhood is considered as the most dreaded phase of life due to strict gender roles, stigma and prevailing traditional customs along with poverty, abuse and lack of social support^17^. Widowhood brings serious economic challenges, and studies have found wealth status to be a strong predictor of health outcomes among older adults^17–19^. However, it is unclear whether economic status after a spouse's death is a primary cause for the psychological distress among older adults.

Marital status and its link with mental health has been explored previously, concluding that unmarried and widowed individuals show higher rates of loneliness, lower life satisfaction, physical ailments, and higher mortality^20^. Globally, about 90 million older adults are estimated to live alone, of which roughly about 60 million are females; still, a great majority of older adults have only been in one union and decide not to marry after the spousal bereavement in older age^21^. Troubled mental state during this phase, such as negative emotions and feelings of emptiness, are challenging for many older adults; as a result, they may tend to withdraw and become unresponsive^22^. However, remarriage in older age is still an exception. Widowed men are observed to remarry at a rate twice as high than female widows^23^. Co-residence with family and community involvement is more common for widows^24,25^. Previous studies have explicitly discussed depression and other psychological illnesses such as dementia in association with population aging, and a few have also reflected on the role of spousal loss in old age^26,27^. The effect of widowhood and co-residence with family together on depression has been observed, but is rather inconclusive; and some studies also suggest that older widowed women who are sick and more depressed might be more likely to co-reside with family^28^.

**Figure 1:**
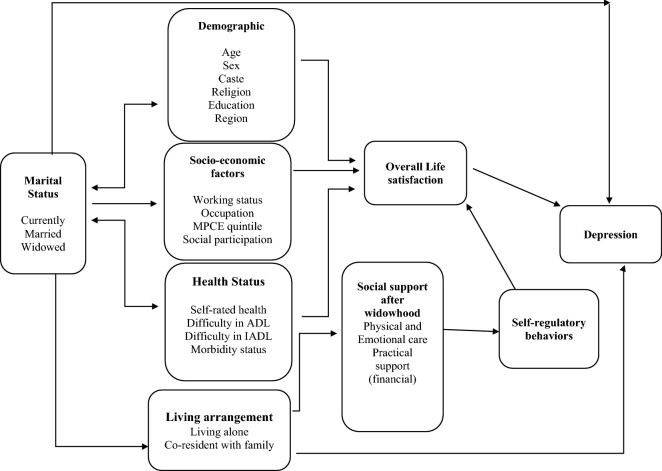
Theoretical framework.

Therefore, the present study attempts to address the literature gap by providing empirical evidence, and to examine the association between marital status and living arrangements with depression among older adults in India. Figure 1 represents the conceptual framework for the present study. Additionally, the study also aims to examine the factors that contribute to the risk of depression among older adults. The study hypothesizes that there is a strong association between widowhood and living alone on depression among older adults.

## Methods

### Data

This study utilizes data from India’s first nationally representative Longitudinal Ageing Study in India (LASI-2017–18), which investigates the health, economics, and social determinants and consequences of population aging in India^29^. The representative sample included 72,250 individuals aged 45 and above and their spouses across all states and union territories of India except Sikkim. The LASI includes 31,464 older adults aged 60 years and above and 6749 oldest-old persons aged 75 years and above. The LASI adopts a multistage stratified area probability cluster sampling design to select the eventual units of observation. Households with at least one member aged 45 and above were taken as the eventual unit of observation. The response rate was 95.6% for the individual interviews and 92.7% in household interviews. The LASI survey provides information on demographics, household economic status, chronic health conditions, symptom-based health conditions, functional and mental health, biomarkers, health care utilization, work, and employment, etc. It enables the cross-state analyses of aging, health, economic status, and social behaviors and has been designed to evaluate the effect of changing policies and behavioral outcomes in India. Detailed information on the sampling frame is available on the LASI WAVE-1 Report.

### Selection of the study sample

**Figure 2:**
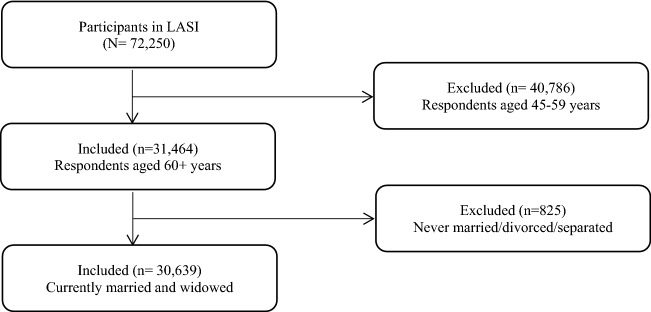
Sample selection criteria.

The effective sample size for the present study was 30,639 older adults. There were no missing values and hence the whole sample was taken into consideration for the analysis. Older adults who were never married/divorced/separated (825 older adults) were dropped from the sample (Fig. 2).

### Variable description

#### Outcome variable

The outcome variable for the study was depression which was coded as 0 for “not diagnosed with depression” and 1 for “diagnosed with depression”. Major depression among the older adults with symptoms of dysphoria, calculated using the CIDI-SF (Short Form Composite International Diagnostic Interview) score of 3 or more on a scale of 0 to 10. The score of less than 3 was categorized as “not diagnosed with depression” and score of 3 and more was categorized as “diagnosed with depression”. This scale estimates a probable psychiatric diagnosis of major depression and has been validated in field settings and widely used in population-based health surveys^29^. The scale was validated for older adults^30^. The questions used to assess depression are presented in appendix.

#### Exposure variables

##### Main exposure variables

Marital status was categorized as currently married and widowed. Living arrangement was categorized as co-residing, which includes living with a spouse, with children, or living with others; and living alone.

##### Other exposure variables

Age was categorized as young old (60–69 years), old-old (70–79 years), and oldest-old (80 + years). Sex was categorized as male and female. Educational status was categorized as no education/primary not completed, primary, secondary and higher. Working status was categorized as currently working, retired, and not working^19^. Social participation was categorized as no and yes. Social participation was measured through the question, “Are you a member of any of the organizations, religious groups, clubs, or societies”? The response was categorized as no and yes. Life satisfaction among older adults was assessed using the questions a. In most ways, my life is close to ideal; b. The conditions of my life are excellent; c. I am satisfied with my life d. So far, I have got the important things I want in life; e. If I could live my life again, I would change almost nothing. The responses were categorized as strongly disagree, somewhat disagree, slightly disagree, neither agree nor disagree, slightly agree, somewhat agree, and strongly agree. Using the responses to the five statements regarding life satisfaction, a scale was constructed. The categories of the scale are ‘low satisfaction (score of 5–20), ‘medium satisfaction’ (score of 21–25), and ‘high satisfaction’ (score of 26–35). Self-rated health was coded as good which includes excellent, very good, and good, whereas poor, includes fair and poor.

Difficulty in ADL (Activities of Daily Living) was coded as no and yes^31^. Activities of Daily Living (ADL) is a term used to refer to normal daily self-care activities (such as movement in bed, changing position from sitting to standing, feeding, bathing, dressing, grooming, personal hygiene, etc.) The ability or inability to perform ADLs is used to measure a person’s functional status, especially in the case of people with disabilities and the elderly. Difficulty in IADL (Instrumental Activities of Daily Living) was coded as no and yes^31^. Activities of daily living that are not necessarily related to the fundamental functioning of a person, but they let an individual live independently in a community. These tasks are necessary for the independent functioning of older adults in the community. Morbidity status was categorized as 0 “no morbidity”, 1 “any single morbid condition”, and 2 + “co-morbidity”. Morbidity was assessed by self-report where the interviewer asked the participant whether any health professional ever diagnosed them with the following diseases? The diseases were a. Hypertension or high blood pressure b. Diabetes or high blood sugar c. Cancer or a malignant tumour d. Chronic lung disease such as asthma, chronic obstructive pulmonary disease/Chronic bronchitis or other chronic lung problems, e. Chronic heart diseases such as Coronary heart disease (heart attack or Myocardial Infarction), congestive heart failure, or other chronic heart problems f. Stroke g. Arthritis or rheumatism, Osteoporosis or other bone/joint diseases h. Any neurological or psychiatric problems i. High cholesterol. All the morbidities were self-reported^32^.

The monthly per capita consumption expenditure (MPCE) quintile was assessed using household consumption data. Sets of 11 and 29 questions on the expenditures on food and non-food items, respectively, were used to canvas the sample households. The variable was then divided into five quintiles, i.e., from poorest to richest. Religion was coded as Hindu, Muslim, Christian, and Others. Caste was recoded as Scheduled Tribe (ST), Scheduled Caste (SC), Other Backward Class (OBC), and others. The ST includes a group of the population that is socially segregated and have a financially/economically low status as per Hindu caste hierarchy. The SC and ST are among the most disadvantaged socio-economic groups in India. The OBC is the group of people who were identified as “educationally, economically and socially backward”. The OBC is considered low in the traditional caste hierarchy but somewhere above the boundary of the most disadvantaged groups. The “other” caste category is identified as having higher social status^33,34^. Place of residence was categorized as rural and urban. The region was coded as North, Central, East, Northeast, West, and South.

### Statistical analysis

In this study, descriptive statistics and bivariate analysis has been performed to determine the prevalence of depression by socio-economic status and other factors in the country. A Chi-square test was used to check the significance level for the bivariate association. Further, binary logistic regression analysis^35^ was conducted to study the association between marital status and living arrangements with depression among older adults. The results are presented in the form of an odds ratio (OR) with a 95% confidence interval (CI). All methods were performed in accordance with the relevant guidelines and regulations. In model-3, interaction effects were observed for marital status and living arrangement with depression among older adults in India. Model-1 was an unadjusted model whereas Model-2 provided the adjusted estimates. Model-3 provides the interaction estimates which are adjusted for all other covariates. In model-3 the interaction effects (*Marital status # Living arrangement*) were observed therefore the independent effect of marital status and living arrangement were estimated. Model-1 was controlled for marital status and living arrangement; Model-2 and 3 was controlled for marital status, living arrangement, age, sex, education, working status, social participation life satisfaction, self-rated health, difficulty in ADL, difficulty in IADL, morbidity status MPCE quintile, religion, caste, place of residence, region.

The regression diagnostics for heteroscedasticity^36^, multicollinearity^37^, and outliers were performed via computation of variance inflation factors (VIFs) and visual inspection of residual plots for the regression models. The cut-off of 10 or more was considered unacceptable; i.e., if the score was 10 or more, then the variables are expected to have multicollinearity between them. The complex survey design effects were adjusted by using STATA *svyset* and *svy* commands. The whole statistical analyses were performed by using STATA version 14^38^.

## Results

Background characteristics of the eligible respondents are presented in Table 1. Analysis indicated that around sixty-three percent of the older adults in India were currently married. A majority of the older Indian adults (94%) were co-residing with their families. As far as education is concerned, the majority of the older adults in the country were uneducated or had not completed primary education (68%), and one-fourth of them had primary or secondary level of education, and only seven percent of them had the higher education.

**Table 1:** Socio-demographic profile of older adults in India.

Background characteristic	Sample	Percentage
**Marital status**		
Currently married	19,302	63.0
Widowed	11,337	37.0
**Living arrangement**		
Co-residing	28,990	94.6
Living alone	1,649	5.4
**Age**		
Young-old	17,902	58.4
Old-old	9,287	30.3
Oldest-old	3,450	11.3
**Sex**		
Male	14,502	47.3
Female	16,137	52.7
**Education**		
No education/primary not completed	20,815	67.9
Primary completed	3,421	11.2
Secondary completed	4,258	13.9
Higher and above	2,144	7.0
**Working status**		
Working	9,387	30.6
Retired	13,127	42.8
Not working	8,126	26.5
**Social participation**		
No	29,263	95.5
Yes	1,376	4.5
Life satisfaction*		
Low	9,444	31.9
Medium	6,615	22.3
High	13,548	45.8
Self-rated health*		
Good	15,451	51.5
Poor	14,567	48.5
Difficulty in ADL*		
No	23,263	76.2
Yes	7,253	23.8
Difficulty in IADL*		
No	15,793	51.8
Yes	14,723	48.3
**Morbidity status**		
0	14,333	46.8
1	8,949	29.2
2 +	7,357	24.0
**MPCE quintile**		
Poorest	6,614	21.6
Poorer	6,660	21.7
Middle	6,421	21.0
Richer	5,906	19.3
Richest	5,038	16.4
**Religion**		
Hindu	25,181	82.2
Muslim	3,480	11.4
Christian	861	2.8
Others	1,116	3.6
**Caste**		
Scheduled Caste	5,827	19.0
Scheduled Tribe	2,496	8.2
Other Backward Class	13,783	45.0
Others	8,534	27.9
**Place of residence**		
Rural	21,639	70.6
Urban	9,000	29.4
**Region**		
North	3,887	12.7
Central	6,440	21.0
East	7,316	23.9
Northeast	912	3.0
West	5,275	17.2
South	6,809	22.2
Total	30,639	100.0

*The sample may differ due to missing cases; ADL: Activities of Daily Living; IADL: Instrumental Activities of Daily Living.

**Figure 3:**
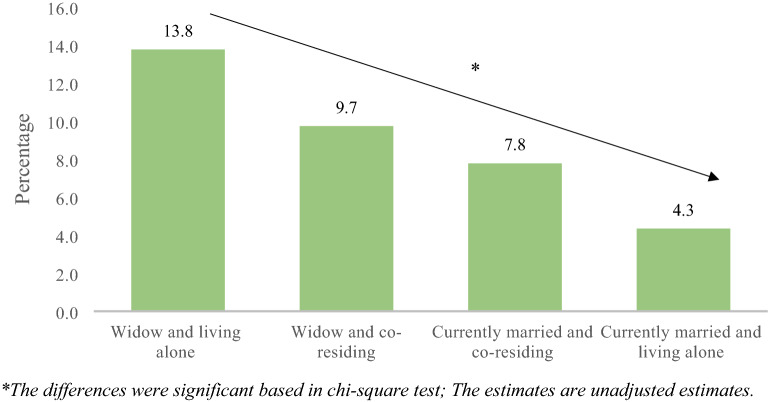
Percentage of older adults suffering from depression by their marital status and living arrangement.

Figure 3 represents the percentage of older adults suffering from depression by their marital status and living arrangement. It was revealed that about 13.8% of older adults who were widowed and living alone suffered from depression in reference to an older adult who was a widow but co-reside (9.7%), currently married, and co-residing (7.8%), and currently married living alone (4.3%). The differences were significant based on the chi-square test.

Table 2 presents the proportion of older adults suffering from depression by background characteristics in the country. Overall, around nine percent of the older adults in the country were suffering from depression. As evident from the data, about 10.3% of the widowed older adults were suffering from depression (currently married: 7.8%). Nearly 13.6% of the older adults who were living alone were suffering from depression whereas around 8.4% of the respondents who were residing with someone were suffering from depression.

**Table 2:** Percentage of older adults suffering from depression by their background characteristic in India.

Background characteristic	%	*P* value
**Marital status**		< 0.001
Currently married	7.8	
Widowed	10.3	
**Living arrangement**		< 0.001
Co-residing	8.4	
Living alone	13.6	
**Age**		0.207
Young-old	8.5	
Old-old	8.4	
Oldest-old	10.9	
**Sex**		< 0.001
Male	7.5	
Female	9.7	
**Education**		< 0.001
No education/primary not completed	9.6	
Primary completed	8.1	
Secondary completed	6.1	
Higher and above	5.8	
**Working status**		< 0.001
Working	7.8	
Retired	10.0	
Not working	7.7	
**Social participation**		< 0.001
No	8.8	
Yes	6.6	
**Life satisfaction**		< 0.001
Low	13.2	
Medium	7.8	
High	6.0	
**Self-rated health**		< 0.001
Good	4.7	
Poor	13.0	
**Difficulty in ADL**		< 0.001
No	6.7	
Yes	15.5	
**Difficulty in IADL**		< 0.001
No	5.6	
Yes	12.1	
**Morbidity status**		< 0.001
0	7.2	
1	8.6	
2 +	11.9	
**MPCE quintile**		< 0.001
Poorest	8.9	
Poorer	8.0	
Middle	8.1	
Richer	8.8	
Richest	10.1	
**Religion**		< 0.001
Hindu	8.6	
Muslim	9.6	
Christian	7.5	
Others	8.5	
**Caste**		< 0.001
Scheduled Caste	10.0	
Scheduled Tribe	5.0	
Other Backward Class	9.3	
Others	7.9	
**Place of residence**		< 0.001
Rural	9.7	
Urban	6.3	
**Region**		< 0.001
North	6.8	
Central	14.6	
East	8.3	
Northeast	5.7	
West	7.6	
South	5.9	
Total	8.7	

%: Percentage; ADL: Activities of Daily Living; IADL: Instrumental Activities of Daily Living; p-value based on chi-square test.

Table 3 shows the results obtained from the logistic regression analysis of the socio-economic status and lifestyle determinants of depression among older adults. Model-1 represents unadjusted estimates and reveals that older adults who were widowed had higher odds to suffer from depression in reference to those older adults who were currently married [UOR: 1.41; CI 1.29, 1.55]. Older adults who were living alone had higher odds to suffer from depression in reference to those older adults who were co-residing [UOR: 1.26; CI 1.05, 1.51]. In model-2, it was found that widowed older adults were 34% significantly more likely to be depressed than currently married older adults [AOR: 1.34, CI 1.2–1.49]. Similarly, respondents who were living alone were 16 percent significantly more likely to be depressed in comparison to older adults who were co-residing [AOR: 1.16; CI 1.02, 1.40]. Model-3 represents the interaction effect of marital status along with living arrangement on depression among older adults. Older adults who were widowed and living alone were 56 percent significantly more likely to suffer from depression [AOR: 1.56; CI 1.28,1.91] in reference to older adults who were currently married and co-residing. Even older adults who were widowed and co-residing 33 percent significantly more likely to suffer from depression [AOR: 1.33; CI 1.19, 1.48] in reference to older adults who were currently married and co-residing.

**Table 3:** Logistic regression estimates for older adults suffering from depression by their background characteristic in India.

Background characteristic	Model-1	Model-2	Model-3
	UOR (95% CI)	AOR (95% CI)	AOR (95% CI)
**Marital status**			
Currently married	Ref	Ref	
Widowed	1.41*(1.29,1.55)	1.34*(1.2,1.49)	
**Living arrangement**			
Co-residing	Ref	Ref	
Living alone	1.26*(1.05,1.51)	1.16*(1.02,1.40)	
**Age**			
Young-old		Ref	Ref
Old-old		0.79*(0.71,0.88)	0.79*(0.71,0.88)
Oldest-old		0.71*(0.60,0.83)	0.73*(0.62,0.83)
**Sex**			
Male		Ref	Ref
Female		1.08(0.96,1.21)	1.08(0.95,1.21)
**Education**			
No education/primary not completed		0.95(0.76,1.19)	0.95(0.76,1.19)
Primary completed		1.01(0.79,1.29)	1.01(0.79,1.29)
Secondary completed		0.95(0.75,1.2)	0.95(0.75,1.2)
Higher and above		Ref	Ref
**Working status**			
Working		Ref	Ref
Retired		0.95(0.84,1.07)	0.95(0.84,1.07)
Not working		0.77*(0.66,0.89)	0.77*(0.66,0.89)
**Social participation**			
No		0.91(0.73,1.12)	0.91(0.73,1.12)
Yes		Ref	Ref
**Life satisfaction**			
Low		2.16*(1.93,2.41)	2.16*(1.93,2.41)
Medium		1.33*(1.17,1.51)	1.33*(1.17,1.51)
High		Ref	Ref
**Self-rated health**			
Good		Ref	Ref
Poor		2.18*(1.96,2.42)	2.18*(1.96,2.42)
**Difficulty in ADL**			
No		Ref	Ref
Yes		1.71*(1.53,1.91)	1.71*(1.53,1.91)
**Difficulty in IADL**			
No		Ref	Ref
Yes		1.56*(1.4,1.74)	1.56*(1.39,1.73)
**Morbidity status**			
0		Ref	Ref
1		1.20*(1.07,1.35)	1.21*(1.07,1.35)
2 +		1.53*(1.36,1.73)	1.53*(1.36,1.73)
**MPCE quintile**			
Poorest		0.76*(0.65,0.89)	0.76*(0.65,0.89)
Poorer		0.75*(0.64,0.87)	0.75*(0.64,0.87)
Middle		0.67*(0.57,0.78)	0.67*(0.57,0.77)
Richer		0.85*(0.74,0.99)	0.85*(0.74,0.98)
Richest		Ref	Ref
**Religion**			
Hindu		Ref	Ref
Muslim		1.07(0.93,1.24)	1.07(0.93,1.24)
Christian		0.94(0.74,1.22)	0.95(0.74,1.21)
Others		1.15(0.92,1.44)	1.16(0.92,1.45)
**Caste**			
Scheduled Caste		1.09 (0.94,1.26)	1.09 (0.94,1.26)
Scheduled Tribe		0.63*(0.52,0.77)	0.63*(0.52,0.77)
Other Backward Class		1.19*(1.06,1.35)	1.19*(1.06,1.35)
Others		Ref	Ref
**Place of residence**			
Rural		Ref	Ref
Urban		1.24*(1.11,1.39)	1.24*(1.11,1.39)
**Region**			
North		Ref	Ref
Central		2.15*(1.84,2.51)	2.15*(1.84,2.51)
East		1.00(0.86,1.17)	1.00(0.86,1.17)
Northeast		0.63*(0.5,0.81)	0.63*(0.5,0.81)
West		1.23*(1.04,1.46)	1.23*(1.04,1.46)
South		0.64*(0.54,0.76)	0.64*(0.54,0.76)
**Marital status # Living arrangement**			
Widowed # co-residing			1.33*(1.19,1.48)
Currently married # co-residing			Ref
Currently married # living alone			0.54(0.12,2.32)
Widowed # living alone			1.56*(1.28,1.91)

^#^: Interaction; Ref: Reference; *if *p* < 0.05; AOR: Adjusted odds ratio; CI: Confidence interval; ADL: Activities of Daily Living; IADL: Instrumental Activities of Daily Living; Model-1 was controlled for marital status and living arrangement; Model-2 and 3 was controlled for marital status, living arrangement, age, sex, education, working status, social participation life satisfaction, self-rated health, difficulty in ADL, difficulty in IADL, morbidity status MPCE quintile, religion, caste, place of residence, region.

In model-2, it was additionally found that older adults who were not working were found to be negatively associated with depression. A lower level of life satisfaction was found to be significantly positively associated with depression [AOR: 2.16, CI 1.93–2.41]. Respondents who reported the life satisfaction as ‘low’ and ‘medium’ were 2.16 [AOR: 2.16, CI 1.93–2.41] times and 1.33 [AOR: 1.33, CI 1.17–1.51] times more likely to have depression in comparison to those who reported their life satisfaction level as ‘high’ respectively. Further, respondents who rated their health as poor were 2.18 times significantly more likely to be depressed than those who reported their health to be good [AOR: 2.18, CI 1.96–2.42]. The likelihood of getting depressed was higher among those who had difficulty with ADL activities than those who did not have any difficulty with ADL activities [AOR: 1.71, CI 1.53–1.91]. Similarly, older adults who had difficulty in IADL 56% significantly more likely to be depressed than older adults who do not have difficulty with IADL activities [AOR: 1.56, CI 1.4–1.74]. Having two and above morbidity conditions had a significant positive association with the prevalence of depression [AOR: 1.53, CI 1.36–1.73]. Older adults from the poorest wealth quintile had lower odds to suffer from depression in reference to older adults from richest wealth quintile [AOR: 0.76; CI 0.65–0.89]. Older adults from OBC had higher odds of suffering from depression in reference to older adults from others category [AOR: 1.19; CI 1.06,1.35]. Older adults from an urban place of residence had higher odds to suffer from depression in reference to older adults from rural counterparts [AOR: 1.24; CI 1.11–1.39].

## Discussion

Several epidemiological studies suggest that the non-co-residential living arrangements of older adults are often associated with the prevalence of depressive symptoms^7,39,40^. Supporting this, our study produced similar findings, with 10.3 percent and 13.6 percent of older adults who were widowed and lived alone, respectively (against 7.8 and 8.4 percent of those currently married and co-residing) reporting severe depressive symptoms as measured by the CIDI-SF 10. The current study, using the first large nationally representative survey data of LASI, included a wide range of potential explanatory variables and adds to the few studies that have been conducted in low-income countries. Previous studies in middle- and high-income countries as well as some low-income countries like China have also observed a significant association between marital status, living arrangements and mental health among older adults^41–44^.

In our multivariable models, living arrangement and marital status were found to be significantly associated with depressive symptoms. These findings are parallel to studies that identified widowhood and solo living as risk factors to poor mental health among older adults in general^45–48^ and those who are oldest-old in particular^48–51^. Also, the interactive effect of marital status and living arrangement with depression was concordant with a recent study in Japan that has shown the adverse impact of both widowhood and living alone on depressive symptoms^52^. The possible explanation for the negative effect of widowhood on psychological wellbeing may be the increased stress due to spousal loss that results in reduced emotional support and lack of financial support^53,54^. Also, as documented, solo-living older individuals are less likely to have a family or close friends to talk to and are thus more likely to have a strong sense of loneliness resulting in depression^55–57^. On the other hand, contrary to other population-based studies^58,59^, older age was found to be inversely associated with depression among older adults in the present study. It can be explained in part by older persons’ reduced levels of emotional reactivity by increasing age leading to lesser reporting of symptoms of extreme distress or depression compared to their younger counterparts^60^. However, the increased depression associated with widowhood and solo living in older adults in our study calls for special attention from policy makers since older Indian adults are left with no or minimum social security and support.

Interestingly, the older adults who were currently in a marital union and lived alone in the present study had lower likelihood of late-life depression, as compared to married and co-residing individuals (Fig. 3). This is contrary to multiple studies showing that solo living among older individuals may lead to mental illnesses^22,61^. This inconsistent finding could partially be explained by the self-selection for individuals who enjoy living alone. The relatively smaller sample of older people who lived alone in our study could also have resulted in the lower odds of depression in solo living individuals due to power effects. Furthermore, consistent with existing literature, the factors identified with a linkage to the increased rates of depression in later life in this study include sex^40,44^, socio-economic resources^62^, and health status^63,64^. The results of the study revealed that older females and those who faced poor self-rated health, low ADL and IADL, and a higher number of diseases reported a higher rate of depressive symptoms. Findings are also in line with the evidence suggesting that the cumulative impacts of disadvantages may increase the risk for negative mental health outcomes as people grow older^65,66^. Consistent with past studies that have found urban–rural differences in terms of psychological wellbeing and depressive symptoms, the current results show that rural living older adults have an increased risk of depression^67–69^. Further, lower social class may contribute to the psychological distress among older adults due to their lack of household economic resources and financial strain^70^, which is supported by current finding of higher prevalence of depression among older adults from OBC category. However, the current study also observed that people from ST are at lower risk of late-life depression in comparison to ‘other’ caste category which includes castes that are considered socioeconomically advantaged. This finding suggests a need for further investigation exploring the variations in mental illnesses among different caste groups in India.

In addition, the significant association of life satisfaction with depression in our study was consistent with previous studies that have shown that older individuals who had greater life dissatisfaction were more likely to suffer from depression and suggested that such a subjective status can be promoted by enhancing their social and economic circumstances^71,72^. Nonetheless, the association of higher household economic status with an increased risk of depression among older adults in our study is in variance with the existing literature that shows that economic status is negatively associated with late-life depression^73–75^. Further studies are required to better understand the risk of developing depression among higher socio-economic groups.

There are several limitations of the present study to be acknowledged. The major limitation is the cross-sectional design of the study, eliminating the drawing of causal inferences among variables. Indeed, it is important to consider that some individuals may become lonelier because they feel depressed or lack energy. Moreover, the study is limited to older adults who are widowed or currently in a marital union. Thus, the exclusion of those who are never married/ divorced/ separated may affect the findings of the study in terms of the association of marital status with late-life depression. Importantly, in the study sample, only 5.4% of the older adults were found to be living alone. This may have led to underestimation of the association, lack of power or type II error and could also have impacted the current findings. Notwithstanding these limitations, the present study's findings provide empirical support to the body of literature that highlights the vulnerability of widowed older adults living alone.

## Conclusion

The findings of the study highlight that being in a marital union and co-residential living arrangements are an essential part of mental well-being in later years of life. The study synthesized its finding with theoretical understandings of vulnerability. As in this study, older widowed women who were exposed to vulnerabilities like lower socioeconomic conditions, poor health status etc. were mainly at a higher risk of depression. Importantly, the information deriving from this study can be used to target outreach programs and service delivery for the older adults who are living alone or widowed and suffering from depression.

## Supplementary Information

Supplementary Information.

## Data Availability

The study uses secondary data which is available on reasonable request through https://www.iipsindia.ac.in/content/lasi-wave-i.
