# Supplementary material for: The association of widowhood and living alone with depression among older adults in India
Source: Sci Rep. 2021 Nov 4;11:21641. doi: 10.1038/s41598-021-01238-x (PMC8568934; doi:10.1038/s41598-021-01238-x)
Supplement: Supplementary file 1 — Supplementary Information. [file 41598_2021_1238_MOESM1_ESM.docx]

**Appendix**

The questions which were used to assess the depression were as follow:-

1. During the last 12 months, was there ever a time when you felt sad, blue, or depressed for two weeks or more in a row?
2. Please think of the two weeks during the last 12 months when these feelings were worst. During that time, did the feelings of being sad, blue, or depressed usually last all day long, most of the day, about half the day, or less than half the day?
3. During those two weeks, did you feel this way every day, almost every day, or less often than that?
4. Did you lose interest in most things?
5. Did you ever feel more tired out or low in energy than is usual for you?
6. Did you lose your appetite?
7. During the same two-week period, did you have a lot more trouble concentrating than usual?
8. People sometimes feel down on themselves and no good or worthless. During those two weeks, did you feel this way?
9. Did you think a lot about death – either your own, someone else’s, or death in general – during those two weeks?
10. Did you have more trouble falling asleep than you usually do during those two weeks?
